# A systematic review of the impacts of remote patient monitoring (RPM) interventions on safety, adherence, quality-of-life and cost-related outcomes

**DOI:** 10.1038/s41746-024-01182-w

**Published:** 2024-07-18

**Authors:** Si Ying Tan, Jennifer Sumner, Yuchen Wang, Alexander Wenjun Yip

**Affiliations:** 1 Alexandra Research Centre for Healthcare In The Virtual Environment (ARCHIVE), Alexandra Hospital, National University Health System, Singapore, Singapore; 2 Saw Swee Hock School of Public Health, National University of Singapore, Singapore, Singapore; 3 School of Computing, National University of Singapore, Singapore, Singapore

**Keywords:** Health care, Interdisciplinary studies

## Abstract

Due to rapid technological advancements, remote patient monitoring (RPM) technology has gained traction in recent years. While the effects of specific RPM interventions are known, few published reviews examine RPM in the context of care transitions from an inpatient hospital setting to a home environment. In this systematic review, we addressed this gap by examining the impacts of RPM interventions on patient safety, adherence, clinical and quality of life outcomes and cost-related outcomes during care transition from inpatient care to a home setting. We searched five academic databases (PubMed, CINAHL, PsycINFO, Embase and SCOPUS), screened 2606 articles, and included 29 studies from 16 countries. These studies examined seven types of RPM interventions (communication tools, computer-based systems, smartphone applications, web portals, augmented clinical devices with monitoring capabilities, wearables and standard clinical tools for intermittent monitoring). RPM interventions demonstrated positive outcomes in patient safety and adherence. RPM interventions also improved patients’ mobility and functional statuses, but the impact on other clinical and quality-of-life measures, such as physical and mental health symptoms, remains inconclusive. In terms of cost-related outcomes, there was a clear downward trend in the risks of hospital admission/readmission, length of stay, number of outpatient visits and non-hospitalisation costs. Future research should explore whether incorporating intervention components with a strong human element alongside the deployment of technology enhances the effectiveness of RPM. The review highlights the need for more economic evaluations and implementation studies that shed light on the facilitators and barriers to adopting RPM interventions in different care settings.

## Introduction

Remote patient monitoring (RPM) technologies are gaining traction in healthcare due to the acceleration of technology development and application of artificial intelligence (AI)^1^. The global RPM market is projected to expand rapidly in the next few years, with a compound annual growth rate of 18.9% projected between 2021 and 2028^2^. In brief, RPM interventions involve the use of connected electronic tools to record personal health data outside the traditional care setting so that they can be reviewed by a provider at a different location^3^. There are many core technologies and architectures that enable RPM interventions, which include various kinds of sensors, internet of things (IoT) devices, networking, data centres, cloud computing and blockchain technologies^1,4^. More recently, the rise of Industry 4.0 has also seen the widespread application of AI, enabling advanced data analytics such as predicting when a patient’s health is declining^4^.

RPM interventions have been widely adopted across various patient groups and clinical settings, according to the literature. At the earliest life stage, RPM interventions have been deployed to monitor vital signs such as temperature, pulse rate and pulse oxygen concentration of ill or premature newborn infants that require constant monitoring in the neonatology ward to prevent subsequent adverse events^5^. Towards the end of the life cycle, advances in digital health technologies in geriatric care have given rise to a variety of physical and chemical wearable sensors, wearable sensing platforms and various smart home digital systems to monitor the health of older people while enabling them to maintain a high level of independence^6^. For adults, various RPM interventions have been designed to monitor patients with various physical and mental health challenges such as chronic obstructive pulmonary disease (COPD)^7,8^, hip osteoarthritis and arthroplasties^9^, cancers^10^, chronic diseases such as diabetes^11^, those who undergo home dialysis^12^, neuro-psychiatric conditions^13^ and more recently, COVID-19 patients who were discharged home from the hospital^14^. Despite being perceived as promising to facilitate care transition from hospital to home, evidence of RPM interventions deployed on patients with acute conditions such as cardiovascular diseases are mixed in terms of the ability of the interventions to reduce the risk of hospitalisations^15,16^. This is likely due to heterogeneity in the stages of the disease as well as the severity of the conditions examined. In terms of subjective outcomes, RPM interventions have demonstrated positive outcomes with studies reporting enhanced patient engagement and patient experience^10,11^. RPM interventions have also shown promise in reduction in the number of hospitalisation days^8,10^, potentially resulting in health cost reduction in the longer run.

Although a growing body of evidence evaluating various RPM interventions has emerged in recent years, the evidence is highly heterogeneous, resulting in the lack of conclusive insights from the consolidation of the evidence. For instance, a meta-analysis conducted by Noah et al.^17^ synthesised 27 randomised controlled trials (RCTs) on wearable biosensors but found limited clinical impacts compared to usual care. A more focused review by Iqbal et al.^18^ examining the impacts of digital sensor alerting systems in remote monitoring reported 9.6% mean decrease in hospitalisation and 3% mean decrease in all-cause mortality. In terms of the cost-effectiveness of RPM interventions, a meta-analysis on RCTs conducted by Klersy et al.^19^ assessing the cost-effectiveness and the cost-utility of RPM on multidisciplinary heart failure management when compared with the usual care approach found significantly lower number of hospitalisations among those who received RPM interventions but no difference in length-of-stay as compared to those who received standard care. The meta-analysis highlighted that the lack of prospectively and uniformly collected economic data was hindering more solid claims to be made. As such, a systematic review and meta-analysis to examine a broader scope of safety, clinical outcomes, quality of life and cost-related outcomes of various RPM interventions is timely in lieu of the burgeoning studies evaluating technology-based remote monitoring on various patient groups that emerged in the past decade. In addition, the high-cost burden due to prolonged admission in the hospital setting and the need for care integration also propel the use of RPM interventions to facilitate care transition from a hospital environment to a home setting. To the best of our knowledge, there is hardly any published review that examined the impacts of RPM interventions that aimed to facilitate care transition from a hospital environment to a home setting to date. This model of care is increasingly lauded as a virtual care model that is more efficient, sustainable and desirable^20^. During the COVID-19 pandemic, many health systems around the world were overwhelmed with high occupancy rates in hospitals, especially at the early stage when the world was still fraught with many unknowns and uncertainties with regard to the pathogenesis of the COVID-19 virus. The pandemic has forced health systems around the world to innovate by leveraging RPM interventions to enable continuity in care provision. As the world moves on from the pandemic, reinventing care provision using RPM interventions would require more understanding of their applications and impacts. This review thus aims to address these knowledge gaps. To this end, we pose the following review question: What are the impacts of RPM interventions on clinical outcomes, patient safety, quality of life and cost-related outcomes during the immediate care transition period from hospital to home?

## Results

### Contexts and characteristics of the included studies

**Fig. 1:**
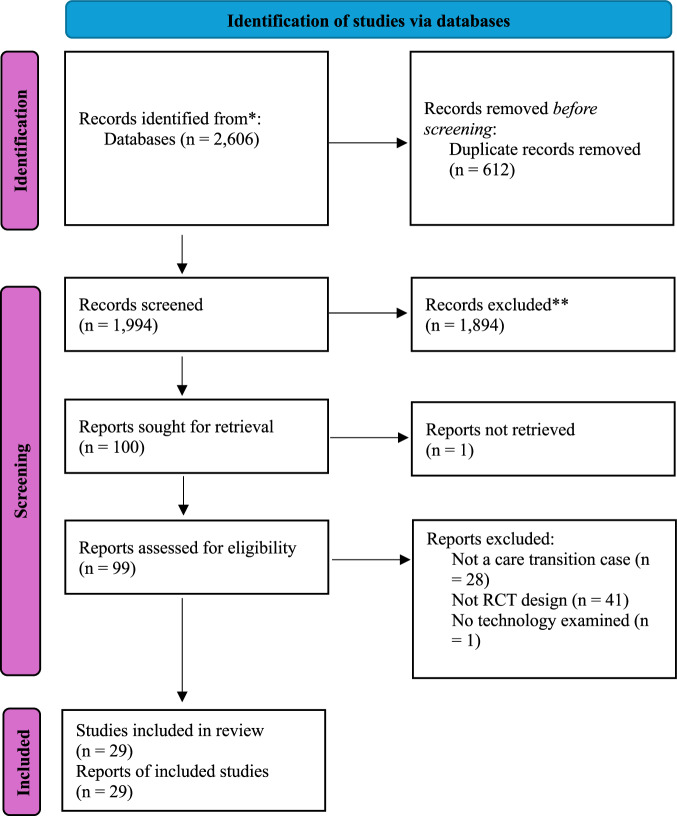
Preferred reporting items for systematic reviews and meta-analyses (PRISMA) flow diagram of the literature search and selection process. The diagram depicts the evidence search, the number of included and excluded studies, and the reasons for article exclusion.

This review identified a total of 30 studies that fulfilled the inclusion criteria and were included in the final synthesis. Figure 1 shows the preferred reporting items for systematic reviews and meta-analyses (PRISMA) flowchart that documents the details of evidence search processes at various stages.

A total of 29 RCTs were included in this review. Studies were conducted in Australia (*n* = 1), Austria (*n* = 1), Brazil (*n* = 1), Canada (*n* = 1), Denmark (*n* = 1), France (*n* = 1), Pakistan (*n* = 1), South Korea (*n* = 1), Spain (*n* = 1), Thailand (*n* = 1), Germany (*n* = 2), Italy (*n* = 2), The Netherlands (*n* = 2), China (*n* = 3) and the US (*n* = 8). Two studies are multi-country, multi-centre studies, with one of them covering France, Italy and Spain and the other one covering the Czech Republic and Germany.

In terms of the population of interest, nearly half of the studies (13 studies) reported the effects of RPM interventions on patients with coronary diseases, including chronic heart failure, acute coronary disease, patients with an implantable cardioverter-defibrillator (ICD) and congestive heart failure. The next most common population examined was patients with lung conditions such as chronic obstructive pulmonary disease (COPD) and lung transplant (four studies). There were three studies on post-surgical patients, two studies examining infants with low birth weight, two studies examining patients with COVID-19 complications, two studies examining patients diagnosed with cancer, one study examining postpartum women, one study examining patients with affective, neurotic and/or behavioural disorder and one study examining internal medicine patients with a wide range of acute, subacute and chronic diseases of different grades and severities (Table 1).

**Table 1:** Contexts and characteristics of the studies included in the review

First author, year (country)	Sample size and mean/median age (Intervention)	Sample size and mean/median age (Control)	RPM technology deployed and intervention components	Control	Duration of the intervention	Target condition or disease	Outcomes
Ahmed et al.^24^ (US)	*N* = 49; mean age = 29.9	*N* = 57; mean age = 29.2	An online, interactive, breastfeeding monitoring system to record breastfeeding and infant output as well as educational resources.	Standard hospital protocol.	1 month	Postpartum women	1°: Breastfeeding rates, 2°: Postpartum depression symptom scores.
Bernocchi et al.^39^ (Italy)	*N* = 56; mean age = 71.0	N = 56; mean age = 70.0	An integrated telerehabilitation home-based programme (Telerehab- HBP). The Telerehab-HBP included remote monitoring of cardiorespiratory parameters, weekly phone-calls by the nurse, and exercise programme, monitored weekly by the physiotherapist.	Standard care programme including medications and oxygen prescription, visits from the general practitioner, and in-hospital check-ups on demand.	4 months	Chronic obstructive pulmonary disease (COPD) and chronic heart failure (CHF)	1°: Six-minute walking test 2°: Median time to hospitalisation/death, dyspnoea, physical activity profile, disability and quality of life.
Blasco et al.^35^ (Spain)	*N* = 102; mean age = 60.6	*N* = 101; mean age = 61.0	Remote monitoring using an automatic sphygmomanometer, a glucose and lipid metre and a cellular phone, regular platform monitoring by cardiologist, individualised short text messaging with recommendations to the patients and lifestyle counselling.	Standard care and lifestyle counselling.	12 months	Acute coronary syndrome (ACS)	1°: Treatment goals for blood pressure 2°: Smoking cessation and body mass index.
Boriani et al.^22^ (France, Italy, Spain, Switzerland)	*N* = 76; mean age = 67.0	*N* = 72; mean age = 68.0	Remote monitoring using CareLink Network (A platform for remote monitoring of implantable cardiac devices, which consists of implantable devices provided with wireless telemetry), CareLink monitor (CLM), and the CareLink website (CW).	Standard care (follow-up without alerts).	12 months	Advanced heart failure	1°: In-hospital visits, 2°: Annual rate of all-cause hospitalisations.
Bouwsma et al.^25^ (The Netherlands)	*N* = 227; mean age = 46.0	*N* = 208; mean age = 45.6	Remote monitoring using a web portal with tailored personalised convalescence advice.	Standard care.	Unspecified	Women undergoing hysterectomy	1°: Duration until full sustainable return to work 2°: The degree of implementation of the care programme was evaluated at the level of the patient, healthcare provider and organisation.
da Silva Schultz et al.^41^ (Brazil)	*N* = 21; mean age = 69.2	*N* = 22; mean age = 69.5	Five telehealth sessions: Telephone follow-up, from a researcher on the 4th, 8th, 12th, 18th and 25th postoperative day.	Standard care.	1 month	Laparoscopic cholecystectomy and hernia repair	1°: Postponed return to work, difficult to move fatigue, perception of recovery, evidence of interrupted healing in surgical site, loss of appetite with nausea, need help to complete self-care, pain, and postoperative sensation.
DeVito Dabbs et al.^49^ (US)	*N* = 99; mean age = 62.0	*N* = 102; mean age = 62.0	A smartphone with custom Pocket PATH programmes to record daily health indicators, graphical displays of trends, and automatic feedback messages advising them to notify the transplant coordinator if health indicators were critical (outside the pre-established parameters) and a toll-free, tech-help line was available.	Standard care (patient education).	12 months	Lung transplant	1°: Self-management behaviour 2°: Self-care agency, rehospitalization, and mortality at home during the first year after transplantation
Ebert et al.^36^ (Germany)	*N* = 200; mean age = 45.0	*N* = 200; mean age = 45.0	A transdiagnostic Internet-based maintenance treatment: a self-management module, asynchronous patient-coach communication, online patient support group, and online- based monitoring of psychopathological symptoms.	Standard hospital protocol.	3 months	Affective, neurotic, and/or behavioural disorders	1°: General psychopathological symptom severity (GPS) 2°: Psychological well-being, depressive symptoms, somatoform complaints, phobic anxiety, interpersonal difficulties, self-efficacy, positive and negative affect, and emotion regulation skills
Fang et al.^40^ (China)	*N* = 33; mean age = 60.2	*N* = 34; mean age = 61.6	Real-time physiological monitoring using a belt strap with a sensor, a smartphone with an application, computer servers and a web portal, booklet given to the participants, weekly phone calls by a physical therapist, and two home visits by a physical therapist.	Standard care (paper-based and self-study CHD booklet and a biweekly outpatient review).	1.5 months	Coronary Heart Disease (CHD)	1°: Six-minute walking test, general health-related quality of life, Fagerstrom Test for Nicotine Dependence, Depression in cardiac patients
Gallagher et al.^38^ (US)	*N* = 20; mean age = 58.0	*N* = 20; mean age = 62.0	A smart phone application and a web portal: A GlowCap® system which includes a pill bottle cap that records the date and time when the bottle is opened (data were sent to the communication hub). Adherence data were reviewed by a licensed clinical social worker on a daily basis during the first 7 days after discharge and weekly thereafter, participants who were non-adherent for two or more days per week were contacted.	Passive monitoring (adherence data were recorded but not actively monitored by the study team).	1 month	Heart failure	1°: Median correct dosing adherence, readmission within 30 days
Geramita et al.^27^ (US)	*N* = 47; mean age = 56.2	*N* = 58; mean age = 56.0	A smartphone with the Pocket PATH application to set reminders for medication-taking and appointments, and record and view graphs for the health indicators that the transplant programme required them to monitor. If health indicator values fell beyond pre-established ranges, Pocket PATH provided decision-support messages instructing them to contact their transplant coordinator.	Standard care.	12 months	Lung transplant	1°: Nonadherence rates in the past month for self-care and lifestyle requirement, nonadherence rates for immunosuppressants and other medications, tobacco use, clinic appointment nonadherence in the past year
Graetz et al.^26^ (US)	*N* = 14; mean age = 56.3	*N* = 15; mean age = 52.9	Smart phone application with daily reminders during the first week post-discharge, every other day for the second week, and once per week in the 3rd and 4th week post-discharge. App contained discharge instructions and symptom tracking, with triggers sent to the clinical team if symptoms were concerning.	Standard care.	1 month	Gynaecological cancer	1°: Mental health composite score
Gray et al.^48^ (US)	*N* = 26; mean age = 27.8 weeks	*N* = 30; mean age = 27.5 weeks	A video conference system with online daily report, messaging centre, baby photos, question & answer, educational and support resources, discharge education, one-time training session on how to use system.	Standard care.	Unspecified	Very low birthweight infants	1°: Length of stay
Guédon-Moreau et al.^37^ (France)	*N* = 158; mean age = 61.4	*N* = 152; mean age = 59.9	ICD (equipped with Biotronik Home Monitoring) linked to a wireless communication system, which automatically transmits diagnostic data and trend analyses between the implanted device and the caregiver on a daily basis.	Ambulatory follow-up only.	27 months	Patients with very low birthweight infants	1°: Non-hospitalisation costs per patient-year, hospitalisation costs per patient-year
Higgins et al.^28^ (Canada)	*N* = 36; mean age = 30.1	*N* = 36; mean age = 30.3	Health mobile application through which were patients responded to questions regarding function, pain, and Quality of Recovery.Range of motion measurements were inputted by physiotherapists during private outpatient appointments. In addition, the surgical-site examination was performed via submitted photos on the app. Pre-operative patient education was also provided.	Pre-operative patient education only.	1.5 months	postoperative anterior cruciate ligament reconstruction	1°: In person clinic visits within 6 weeks of surgery
Hindricks et al.^23^ (Germany and Czech Republic)	*N* = 77; mean age = 63.0	*N* = 78; mean age = 63.0	RM (ICD implant linked to HM) capability (Biotronik, Berlin, Germany) combined with automatic transmission of electrogram data with customisable alerts) with 3-month monitoring interval.	RM (ICD implant linked to HM capability combined with automatic transmission of electrogram data with customisable alerts) with 12-month monitoring interval.	24 months	Patients with implantable cardioverter-defibrillator (ICD)	1°: Number of unscheduled follow-ups per patient-year, total number of scheduled and unscheduled follow-up visits after the 3-month follow-up 2°: Patients remaining after the 3-month follow up, hospitalisations for all causes, hospitalisations for adverse cardiovascular events, patients with ICD therapy delivery
Hisam et al.^43^ (Pakistan)	*N* = 80; mean age = 53.7	*N* = 80; mean age = 51.7	Medically supervised cardiac rehabilitation programme using smart phone application in addition to standard post-ACS care. Individualised psychotherapy during the hospital stay, diurnal mobile texting of standardised messages about healthy lifestyle changes through the app.	Standard post ACS care.	6 months	Acute coronary syndrome	1°: Health related quality of life
Indraratna et al.^32^ (Australia)	*N* = 81; mean age = 61.3	*N* = 83; mean age = 61.7	A smart phone application (TCC app) connected to bluetooth peripheral devices: a digital sphygmomanometer, a digital weighing scale, and a fitness band (Xiaomi MiBand 2). Data available to the clinicians. The app provided three weekly educational push notifications to promote healthy behaviour choices. Customisable limits for BP, pulse rate, and weight gain, which trigger alerts.	Standard care.	6 months	Acute coronary syndrome or heart failure	1°: Number of readmissions at 30 days 2°: Cardiac rehabilitation attendance, cardiac rehabilitation completion
Jakobsen et al.^33^ (Denmark)	*N* = 29; mean age = NA	*N* = 28; mean age = NA	Hospital at home: Touch screen with a Webcam, pulse oximeter, spirometer, thermometer, nebuliser for aerosolized inhalation medication, oxygen compressor, and a medicine box containing antibiotics, prednisone, sedative, beta2 agonists, and anticholinergics. Unscheduled and acute contacts could be made 24/7 by the patient pressing the “call hospital” button on the touch screen.	Standard treatment and care at the hospital.	6 months	COPD	1°: Readmission within 180 days
Li et al.^42^ (China)	*N* = 60; mean age = 58.3	*N* = 60; mean age = 60.8	Videoconferencing (on WeChat app) for post-discharge functional assessment.	Telephone follow-up.	3 months	Stroke	1°: Functional status 2°: Feasibility of using intervention, acceptability of intervention
Li et al.^44^ (China)	*N* = 59; mean age = 49.0	*N* = 60; mean age = 51.0	Unsupervised home-based six-week exercise programme (three to four sessions per week) comprising breathing control and thoracic expansion, aerobic exercise and lower limb muscle strength exercise, delivered via smartphone (ReHab App), and remotely monitored with heart rate telemetry. Weekly teleconsultation with a therapist using WeChat voice calls.	Usual care.	1.5 months	COVID-19 survivors with complaints of dyspnoea	1°: Six-minute walking test 2°: Squat time, pulmonary functions health-related quality of life, physical component score, mental component score, perceived dyspnoea
Pietrantonio et al.^30^ (Italy)	*N* = 56; mean age = NA	*N* = 54; mean age = NA	Telemonitoring device (WINMEDICAL) which allows continuous, real-time vital signs monitoring, automatic calculation of the NEWS (National Early Warning Score) score, and the creation of a personalised alert system for every single patient through a portable device (tablet or phone), a phone call by a nurse, a visit by the nurse 5 days post discharge, the device was removed after five days if patients are stable, continuous follow-up via phone call until day 30.	Usual care.	1 month	Internal medicine patients (comprising a wide range of acute, subacute and chronic diseases of different grades and severities)	1°: Major complications (MC) reduction. 2°: Patients who reached discharge criteria within the 7th day from admission, MC incidence at the conclusion of the standard telemonitoring/clinical monitoring phase, 5 and 30 days after discharge; and conditions predisposing to MC occurrence
Riegel et al.^47^ (US)	*N* = 62; mean age = 59.5	N = 68; mean age = 57.3	Electronic monitoring pill bottle (A Medication Event Monitoring System and financial component (financial reward for medication adherence, deduction of reward for medication non-adherence).	Electronic monitoring pill bottle with no financial compensation.	3 months	ACS	1°: Medication adherence
Scherr et al.^21^ (Austria)	*N* = 54; median age = 67.0	*N* = 54; median age = 65.0	MOBITEL Telemedicine Platform to measure their vital parameters (blood pressure, heart rate, body weight) on a daily basis.	Usual care.	6 months	Heart failure	1°: Cardiovascular mortality or re-hospitalisation 2°: Functional status, length of stay during re-hospitalisations, technical parameters (system availability, cumulative transmission).
Soh et al.^45^ (South Korea)	*N* = 22; mean age = NA	*N* = 22; mean age = NA	Pharmacological treatment and telemedical surveillance using a smart phone application and dashboard (Go-breath app and Go-breath dashboard).	Usual care.	Unspecified	Gastric cancer	1°: Incentive spirometer index (ISI) 2°: System usability scale (SUS)
Somsiri et al.^29^ (Thailand)	*N* = 73; mean age = NA	*N* = 73; mean age = NA	A telehealth programme using a mobile application and usual care (discharge planning and patient education).	Usual care (discharge planning and patient education).	1.5 months	Heart failure	1°: Functional status 2°: Rehospitalization
van Goor et al.^46^ (The Netherlands)	*N* = 31; mean age = 55.1	*N* = 31; mean age = 55.4	RM of medication and oxygen therapy using a mobile health app and telephone monitoring by medical students supervised by internal medicine consultants.	Usual care (medication and oxygen therapy).	1 month	Patients hospitalised with COVID-19	1°: Number of hospital-free days during the 30 days following randomisation 2°: Health care consumption (index hospital length of stay) during the follow-up period, total duration of care under hospital responsibility, and mortality
Weintraub et al.^34^ (US)	*N* = 95; mean age = 69.5	*N* = 93; mean age = 68.5	(i) Automated health monitoring technology (comprising a device with measurement and communication components (ie, transmission of body weight, blood pressure, and heart rate via a standard telephone line to a central server)) and heart failure disease management programme (comprising (i) weekly phone calls by the patient’s nurse manager to review clinical status; (2) a weekly conference, consisting of nurse managers from all clinical sites and a designated physician, to review all actively enroled patients; and (3) 24/7 telephone access to a nurse manager.	Standard heart failure disease management programme only.	3 months	Congestive heart failure	1°: Hospitalisation
Wintrich et al.^31^ (Germany)	(i) RM with no alerts (*N* = 149; mean age = 65.7) (ii) RM with appropriate contacting only (*N* = 113; mean age = 65.8) (iii) RM with inappropriate contacting (*N* = 243; mean age = 66.6)	*N* = 497; mean age = 66.0	Telehealth monitoring with appropriate reaction to the alerts sent. Appropriate contacts had to meet the following criteria: (1) initial telephone contact within 2 working days after fluid index threshold crossings (FTC), (2) follow-up contacts according to study protocol, and (3) medical intervention initiated after FTC due to cardiac decompensation.	Telehealth monitoring with no alerts sent.	18 months	Congestive heart failure	1°: Cardiovascular death or heart failure hospitalisation

Notes: *RM* remote monitoring, *HM* home monitoring.

### Risk of bias assessment

**Fig. 2:**
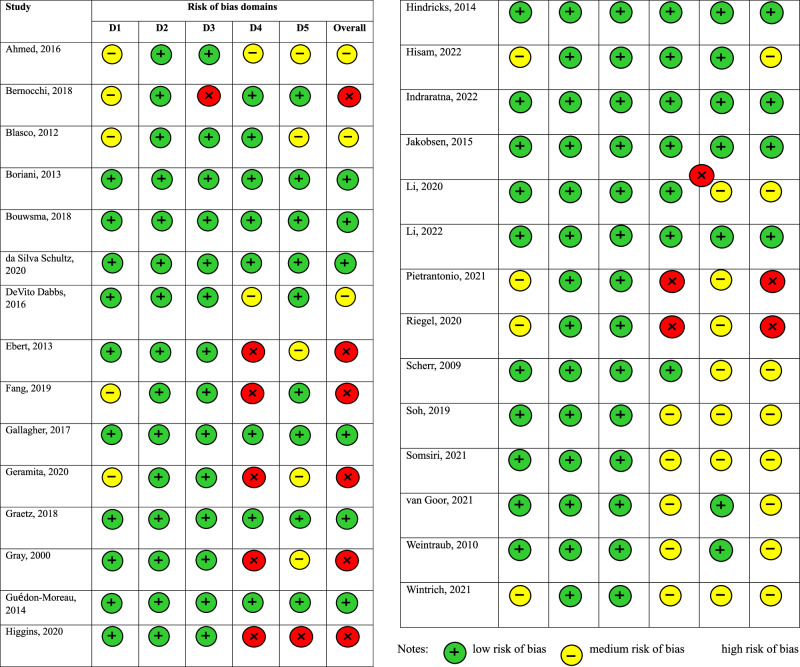
Graphical visualisation of the risk of bias results. We assessed the risk of bias using the revised Cochrane risk-of-bias tool for randomised trials (RoB 2). The tool assesses bias in five domains: risk of bias arising from the randomisation process, risk of bias due to deviations from the intended interventions, risk of bias due to missing outcome data, risk of bias in the measurement of the outcome, risk of bias in the selection of the reported result.

The critical appraisal exercise using the RoB 2 appraisal tool identified nine studies (31.03%) as having overall low risk of bias,11 studies (37.93%) as having overall medium risk of bias and nine studies (31.03%) as having overall high risk of bias. Figure 2 displays the graphical visualisation of the overall risk of bias as well as the risk of bias in each category.

### RPM technologies deployed

We identified seven types of technologies based on their functions, ranging from standard communication tools to computer-based systems, to smartphone applications, to web portal, to augmented clinical devices with monitoring capabilities, to wearables and other clinical tools used to assist remote monitoring. Table 2 provides a summary of the technology types with detailed descriptions and the study examples.

**Table 2:** Types of RPM technologies examined in the review

Types of technology	Description	Examples
Communication tools	Phone call	Bernocchi et al.^39^; Blasco et al.^35^; da Silva Schultz et al.^41^; Li et al.^44^
	Video conference (Webcam or video conference system)	Gray et al.^48^ (video conference system); Jakobsen et al.^33^ (Webcam)
	Social media app (Wechat)	Li et al.^42^; Li et al.^44^
Computer-based systems	System to input patient monitoring data and receive alerts	Ahmed et al.^24^
	System with online daily reports for parents and clinical education	Gray et al.^48^
Smart phone based systems	Smart phone applications	Dabbs et al.^49^; Fang et al.^40^; Geramita et al.^27^; Graetz et al.^26^; Higgins et al.^28^; Hisam et al.^43^; Li et al.^44^; Riegel et al.^47^; Soh et al.^45^; Somsiri et al.^29^; van Goor et al.^46^
Websites	Web portal for data access	Boriani et al.^22^; Bouwsma et al.^25^; Gallagher et al.^38^
Augmented clinical devices with monitoring capabilities	Electronic monitoring pill bottle to send data on opening time (GlowCap® system by Vitality Inc. or CleverCaps® by Compliance Meds Technologies)	Gallagher et al.^38^; Riegel et al.^47^
	Implanted devices (Biotronik)	Guédon-Moreau et al.^37^; Hindricks et al.^23^
Wearables for continuous vital signs and activity monitoring	Specialised monitoring device for basic vital signs (cardiac rate, respiratory rate, blood, peripheral saturation, temperature, and position of the patient) (WINMEDICAL)	Pietrantonio et al.^30^
	Activity monitor	Blasco et al.^35^
	Belt strap with a sensor	Fang et al.^40^
	Fitness band (Xiaomi MiBand 2)	Indraratna et al.^32^
Clinical tools for intermittent monitoring	Sphygmomanometer	Blasco et al.^35^; Indraratna et al.^32^; Scherr et al.^21^
	Weighing scale	Indraratna et al.^32^; Scherr et al.^21^
	Pulse oximeter	Blasco et al.^35^; Jakobsen et al.^33^
	Thermometer	Jakobsen et al.^33^
	Spirometer	Jakobsen et al.^33^
	Glucose and lipid metre	Blasco et al.^35^

Specifically, communication tools refer to information technologies mainly used to assist communication among different parties in different locations. Computer-based systems refer to information systems installed and used on specific computers. Smartphone-based systems are information systems installed and used on specific mobile phones. Websites are webpages accessible from various technological devices through an internet connection. Augmented clinical devices with monitoring capabilities refer to conventional clinical devices that are substantiated with monitoring functions. Wearables for continuous vital signs and activity monitoring capture continuous monitoring data of vital signs and patient activities. Clinical tools for intermittent monitoring are devices that capture episodic patient data.

In terms of technology purpose and function, we identified two general purposes: (i) delivery of existing clinical services remotely, and (ii) delivery of new clinical services. For the first general purpose, we identified seven studies that can be further subdivided into three sub-groups based on their detailed purposes, which include (i) replacing existing monitoring technology with a new one, (ii) replacing in-person follow-up services with remote monitoring, and (iii) replacing inpatient monitoring to enable earlier transition to home or other clinical institutions. For the second general purpose, we identified 20 studies and further classified them into four sub-groups based on their detailed purposes. These include (i) supplementing existing interventions with remote monitoring, (ii) providing new care services which include personalised care, self-management and patient education, (iii) improving patient adherence and patient engagement, and (iv) reducing information asymmetry for caregivers (see Table 3).

**Table 3:** Functional analysis of RPM technologies examined in the review

General purpose	Detailed purposes	Examples
Delivery of existing clinical services remotely	Replace existing monitoring technology with a new one	Li et al.^42^
	Replace in-person follow-up services with remote monitoring	Boriani et al.^22^; DaSelva Schults et al.^41^; Higgins et al.^28^; Hindricks et al.^23^
	Replace inpatient monitoring to enable earlier care transition	Jakobsen et al.^33^; van Goor et al.^46^
Delivery of new clinical services	Supplement existing interventions	Ebert et al.^36^; Pietrantonio et al.^30^; Scherr et al.^21^; Somsiri et al.^29^; Wintrich et al.^31^, Soh et al.^45^
	Provide personalised care, self-management and patient education	Ahmed et al.^24^; Bernocchi et al.^39^; Blasco et al.^35^; Bouwsma et al.^25^; DeVito Dabbs et al.^49^; Geramita et al.^27^; Hisam et al.^43^; Indraratna et al.^32^; Li et al.^44^; Weintraub et al.^34^
	Improve patient adherence and engagement	Fang et al.^40^; Gallagher et al.^38^; Graetz et al.^26^, Riegel et al.^47^
	Reduce information asymmetry of caregivers	Gray et al.^48^; Guédon-Moreau et al.^37^

In our review of RPM technologies, we identified three primary monitoring modes: alert-driven, scheduled, and unscheduled. In alert-driven mode, clinical staff respond to patients following system alerts or proactive patient contact. Contact initiation in alert-driven mode depends on who receives the alert, be it clinical staff or patients. We found 14 studies employing an alert-driven monitoring mode. Of these, 12 studies used system alerts to notify clinical staff of abnormal conditions^21–32^, while two studies sent alerts only to the patients^25,33^. For the technology functioning based on scheduled monitoring mode, the user with the monitor role (usually the clinical staff or family caregivers) played proactive roles: they would adhere to schedules to regularly check patients’ conditions. We identified 11 studies adopting a scheduled monitoring mode^34–44^. On the other hand, four studies adopted unscheduled monitoring mode by performing ad-hoc checks on the monitoring data to detect clinical anomalies^45–48^.

### Impacts of the RPM interventions on patient safety

Three studies documented the impacts of various RPM interventions on patient safety—operationalised as major complication reduction and/or adverse event such as mortality—as the primary outcomes.

#### Major complication reduction and adverse event

Pietratonio et al.^30^ demonstrated that the intervention arm patients who were mostly discharged from the internal medicine ward with a wide range of acute, subacute and chronic diseases of different grades and severities reported fewer major complications, which include rehospitalisation, than control arm patients. Regarding mortality, Scherr et al.^21^ observed lower death risks in telemonitored patients, and Wintrich et al.^31^ noted reduced cardiovascular death risks in chronic heart failure patients using alert-enabled remote monitoring.

### Impacts of the RPM interventions on adherence

In terms of adherence, four studies examined the impacts of RPM interventions on adherence towards medication or lifestyle prescriptions, while one study examined the impacts on the odds of self-monitoring.

#### Adherence to medication or lifestyle prescriptions

Blasco et al.^35^ reported a higher and statistically significant rate of adherence to the exercise routine prescribed to moderate to severe chronic obstructive pulmonary disease (COPD) patients while Geramita et al.^27^ reported statistically significant lower risk to nonadherence to lifestyle requirements for patients who were enroled in RPM intervention as opposed to patients underwent usual care. As for medication adherence, Gallagher et al.^38^ found no significant difference between heart patients who were enroled for RPM intervention as compared to patients who were on passive monitoring. On the other hand, Riegel et al.^47^ found a significant decline in the median adherence towards medication among the control group patients who were not monitored as compared to patients who were monitored with electronic monitoring pill bottles using a medication event monitoring system.

#### Odds of self-monitoring

When examining patients who underwent lung transplant and were discharged with a tracking technology device, DeVito Debbs et al.^49^ reported significantly higher odds of self-monitoring among them when comparing to patients who were not discharged with the same remote monitoring device.

### Impacts of the RPM Interventions on Clinical Outcomes and Quality of Life

14 studies reported the impacts of RPM interventions on clinical and quality of life outcomes which include mobility function or functional statuses^29,39–44^ occurrence of various physical and mental symptoms^26^, breathing outcome^45^, breastfeeding outcome^24^, various postoperative symptoms and sensations^41^ and general psychopathological symptom^36^ as well as duration until return to work^25^.

#### Mobility/functional statuses

Seven studies reported mobility or functional status as its primary outcome for RPM interventions. Bernocchi et al.^39^ reported better mobility outcomes after four months in the form of longer walking distances for rehabilitation patients who were remotely monitored as compared to those who were not remotely monitored. Both Fang et al.^40^ and Hisam et al.^43^ similarly reported significantly better mobility outcomes (measured using the 6-min walking test, SF-36 and SF-12) for coronary heart disease patients who were monitored in real-time with a smartphone app as compared to those who were placed on usual care. Moreover, Fang et al.^40^ also included active and rapid feedback between patients and clinicians as an intervention component alongside the remote monitoring technology. In examining stroke patients monitored with a smartphone application known as WeChat, Li et al.^42^ nevertheless reported no significant change in their functional statuses, measured using the modified Barthel index, when compared with patients who were not remotely monitored using the application. On the other hand, Li et al.^44^ reported better and significant mobility and functional status outcomes among COVID-19 patients with complaints of dyspnoea who were discharged home and monitored using a combination of smartphone application and WeChat voice call as compared to those who were discharged but not remotely monitored using the same technologies. Likewise, Somsiri et al.^29^ reported significantly better functional statuses and increased satisfaction with care scores among patients diagnosed with heart failure and monitored with a smartphone application as compared to those who underwent usual care. DeSilva Schultz et al.^41^ investigated the effects of telehealth in monitoring post-surgical patients who were discharged from the hospital and reported a significantly higher level of independence (measured using a binary question as to whether they need help to complete self-care) among them as compared to patients who were not monitored using telehealth.

#### Occurrence of various physical and mental symptoms

Six studies examined the occurrence of a multitude of physical and mental health symptoms to reflect quality of life outcomes and the evidence is mixed. Ahmed et al.^24^ reported no significant difference in breastfeeding outcomes among women with postpartum depression who were remotely monitored as compared to those who were not remotely monitored. However, de Silva Schultz et al.^41^ also reported a significantly lower incidence of symptom occurrence, such as loss of appetite among post-surgical patients who were monitored with telehealth as compared to patients who were placed on usual care. Ebert et al.^36^ similarly reported significantly lesser symptom deterioration among patients with affective, neurotic and/or behavioural disorders randomised to receive an internet-based maintenance treatment programme, online patient education and online support group as compared to patients who were not supported with the same internet-based programmes. Graetz et al.^26^ also reported a slightly increased but insignificant mental health score among post-surgical patients discharged with a self-monitoring application when compared with patients discharged without a self-monitoring application. In a similar vein, Soh et al.^45^ also reported better but insignificant breathing outcomes (measured using incentive spirometer index) among post-surgical patients placed on remote monitoring technology (Go-breath application and dashboard) as opposed to patients who were not remotely monitored.

#### Duration until return to work

Only one study reported duration until return to work to reflect quality of life. Based on a study conducted in The Netherlands, Bouwsma et al.^25^ reported that women who went through hysterectomy and were placed on follow-up using a web portal after discharge were able to return to work significantly faster than those who were not placed on the same remote monitoring technology.

#### Impacts of the RPM Interventions on cost-related outcomes

A total of 12 studies investigated the impacts of RPM interventions on cost-related outcomes. Among them, 11 studies examined cost-related outcomes in terms of either frequency or risk of hospital admission/readmission^22,31–34,47,48^, length of stay^21^, number of subsequent outpatient visits^23,28^ and number of hospital-free days^46^. Only one study operationalised cost-related outcomes as hospitalisation/non-hospitalisation costs^37^.

#### Hospital admission/readmission outcomes and length of stay

Boriani et al.^22^ showed that in-hospital visits were reduced in the intervention group monitored with a remote monitoring device as compared to the control group patients, and the median delay from device-detected events to clinical decisions made was considerably shorter in the intervention group patients when comparing with the control group. Likewise, Indraratna et al.^32^ also reported that the risk of 30-day readmission was lower among patients who were remotely monitored. Nevertheless, Jakobsen et al.^33^ did not report a significant difference in the risk of 100-day readmission among patients who were remotely monitored and not remotely monitored. Riegel et al.^47^ showed that even though the incidence of rehospitalisation among the intervention group patients monitored with technology was lower, it was not statistically significant when compared with control group patients. Scherr et al.^21^ also documented a significantly shorter length of stay, while Weintraub et al.^34^ and Wintrich et al.^31^ reported a lower risk of hospitalisations among patients monitored with technology as compared to patients who were not monitored with technology. Gray et al.^48^, on the other hand, reported that infants in the intervention groups monitored using teleconferencing technology experienced shorter lengths of stay than the infants in the control group that received usual care, though the result is insignificant.

#### Number of outpatient/follow-up visits

Higgins et al.^28^ reported significantly higher post-surgical outpatient visits among patients who were not remotely monitored as compared to patients who were monitored remotely. Likewise, Hindricks et al.^23^ reported that the number of unscheduled visits was significantly lower among patients who were remotely monitored as compared to patients who were not remotely monitored. In addition, the total number of follow-up visits (both scheduled and unscheduled) was significantly lower among the remotely monitored patient group as well.

#### Number of hospital-free days

van Goor et al.^46^ examined the impact of remote monitoring using a mobile application on patients infected with COVID-19 who were discharged home and found that the mean difference in the number of hospital-free days during the 30 days following randomisation was only 1.7 days less than patients who received standard care. The result was insignificant. However, the index hospital length of stay was 1.6 days shorter (*p* < 0.001) in the intervention group when compared to the control group.

#### Hospitalisation/non-hospitalisation costs

Guédon-Moreau et al.^37^ examined the cost impacts by looking at both the hospitalisation and non-hospitalisation costs per patient-year on patients with very low birthweight infants over a period of 27 months. They found that the mean non-hospitalisation costs per patient-year were significantly lower among patients who were remotely monitored as compared to those who received standard care. In terms of hospitalisation costs, there was no significant difference between patients who were remotely monitored as compared to those who received standard care even though patients who were remotely monitored incurred a slightly lower hospitalisation cost.

Table 4 summarises the results of the impacts of RPM interventions on patient safety, care continuity, quality of life and cost outcomes for all 29 included studies.

**Table 4:** Impacts of RPM interventions on patient safety, adherence, clinical/quality of life and cost-related outcomes

First author, year	Monitoring Mode	Patient safety		Adherence		Clinical/ quality of life outcomes			Cost-related Outcomes			
		Major complications	Adverse event	Adherence to medication or lifestyle prescription	Odds of self-monitoring	Mobility/ functional statuses	Occurrence of various physical and mental symptoms	Duration until return to work	Hospital admission/ readmission/ length of stay	Number of outpatient/ follow-up visits	Number of hospital-free days	Hospital-isation/ non-hospital-isation costs
Pietratonio et al.^30^	AM	Decrease							Decrease			
Scherr et al.^21^	AM		Decrease						Decrease			
Wintrich et al.^31^	AM		Decrease						Decrease			
Blasco et al.^35^	SM			Increase								
Geramita et al.,^27^	AM			Increase								
Gallagher et al.,^38^	SM			Neutral								
Riegel et al.^47^	UM			Increase					Neutral			
Dabbs et al.^49^	AM				Increase							
Bernocchi et al.,^39^	SM					Increase						
Fang et al.,^40^	SM					Increase						
Hisam et al.^43^	SM					Increase						
Li et al.^42^	SM					Neutral						
Li et al.^44^	SM					Increase						
Somsiri et al.^29^	AM					Increase						
Schultz et al. 2020	SM					Increase	Decrease					
Graetz et al.,^26^	AM						Neutral					
Soh et al.^45^	UM						Neutral					
Ahmed et al.^24^	AM						Neutral					
Ebert et al.,^36^	SM						Decrease					
Bouwsma et al.,^25^	AM							Decrease				
Boriani et al.^22^	AM								Decrease			
Indraratna et al.^32^	AM								Decrease			
Jakobsen et al.^33^	AM								Neutral			
Weintraub et al.^34^	SM								Decrease			
Gray et al.,^48^	UM								Neutral			
Higgins et al.^28^	AM									Decrease		
Hindricks et al.^23^	AM									Decrease		
van Goor et al.^46^	UM										Neutral	
Guédon-Moreau et al.,^37^	SM											Neutral (hospital costs); Decrease (Non-hospital cost)

Notes: (i) increase: increase/higher/more; decrease: decrease/lower/lesser; neutral: no effect/effect was insignificant. (ii) The results reflect the directions for the intervention groups as compared to the control groups. (iii) *SM* scheduled monitoring, *UM* Unscheduled monitoring, *AM* alert-driven monitoring.

## Discussion

This review encompasses thirty RCTs investigating seven primary RPM interventions leveraging various technologies, including communication tools, computer-based systems, smartphone apps, web portals, augmented devices, wearables, and standard intermittent monitoring tools. These technologies aim to deliver remote services, supplement on-site interventions, offer personalised care, enhance patient adherence and education, and reduce caregivers’ information asymmetry. Overall, RPM interventions have positively impacted patient safety, evidenced by reduced major complications and adverse events. Outcomes on adherence are rather encouraging as well, with RPM interventions showing an overall upward trend in patient adherence to medication and lifestyle prescription as well as the odds of self-monitoring. In terms of clinical outcomes and quality of life, RPM interventions have shown improvements in the mobility and functional statuses of patients in general. However, the evidence on the risk of various physical and mental symptoms is somewhat mixed. For cost-related outcomes, reduced risks of hospital admission/readmission, length of stay, number of subsequent follow-ups and non-hospitalisation costs are clearly observed. In terms of monitoring mode, there is no stark difference between technologies that utilise alert-driven monitoring mode versus scheduled and unscheduled monitoring modes. Due to the heterogeneity of the interventions and the comparator groups, it is beyond the scope of this review to establish the extent to which other intervention components with strong human elements incorporated alongside the deployment of the technology, such as active phone calls from the healthcare workers, patient training and education and online support groups influence and/or enhance the effects of the RPM interventions. The presence of human elements in terms of provider follow-up, skills training and patient education can affect the overall effects of technological interventions. Follow-up inquiries on the human intervention weaved into the RPM intervention deployment would be pertinent to tease out how much they are augmenting or diminishing the impacts of the whole intervention.

One of the major strengths of this review is the identification and mapping of a wide range of RPM technologies. We searched five academic databases, consulted an information specialist to fine-tune our search strategy, included 30 RCTs and examined a wide range of impacts, from patient safety outcomes to service quality outcomes, to cost outcomes. These evaluations were conducted in 16 countries covering health systems with different developmental stages, signalling the burgeoning pace and relevance of the RPM intervention to deliver effective healthcare and bridge health system gaps, especially in light of the COVID-19 pandemic.

However, the diversity in patient populations, intervention durations, and components across these studies complicate drawing definitive conclusions about RPM’s effects on specific diseases. Also, heterogeneity of the outcome measures derived from this review rule out the possibility of a meta-analysis to be undertaken. It is important to acknowledge that RPM intervention is constantly evolving, and it is possible that we may not have included all pertinent terms in our search process. While we have included respective concepts for ‘remote’ and ‘monitoring’, our search may not have captured the full gamut of the ‘remote monitoring’ concept.

There are a multitude of demographic, system- and individual-level factors that have facilitated the rapid development and adoption of RPM interventions in healthcare. In terms of demographic factors, an ageing population with heightened care needs has been a key driver in the rise in popularity of RPM interventions. This is especially so in recent years whereby the concept of ageing in place is gaining policy attention. RPM interventions are widely regarded as effective nonpharmacological tools that allow physicians to monitor many types of acute and chronic conditions among the older population who require close monitoring but prefer to be tracked in the comfort of their homes without being institutionalised^1^. At the system level, the ballooning healthcare expenditures and insufficient healthcare resources in terms of manpower and service provision also play key roles in the acceleration of RPM intervention adoption^1,5^. However, some argue that RPM may not be cost-effective. For instance, Mecklai et al.^50^ argued that the change of reimbursement rule in the US—the establishment of new billing codes by the Centre of Medicaid Services for Chronic Care RPM in 2019 and a revision in 2020, which enabled reimbursement for the initial setup of RPM interventions and their associated services which include patient education, collection and interpretation of data and treatment management services) may well escalate healthcare expenditures in the health system due to increased RPM intervention uptake. According to the authors’ calculation based on administrative data, a conservative estimate that assumes RPM enrolment and dissemination of the RPM interventions to be limited to patients with multiple chronic conditions alone (which is about 25.4 million patients as of September 2020), and placing a cap of uptake level at 50%, could translate into maximum annual cost per patient enrolled into the RPM programme at US$1,460, with annual health expenditures exceeding US$18 billion^50^. At the individual level, social and behavioural factors such as influences from family and friends as well as the duration of using the technology play important roles in influencing technology acceptance towards the use of technologies for health tracking purpose^51,52^. Policymakers and healthcare administrators can take note of these facilitators to test and deploy RPM interventions that could augment and supplement the functional roles of human healthcare workers while addressing the health needs of different segments of populations based on their unique contextual conditions. It is nonetheless important for these technologies to be subjected to constant evaluation not only to assess their impacts but also their acceptability and usability.

It is equally important for policies and practices around RPM interventions to be vigilant of the barriers to technology adoption, including the downsides, risks and unintended consequences that technology can bring. For instance, huge financial commitment entailing high start-up costs and ongoing operational costs, shortfalls in technical skills, which include lack of training, technical support and infrastructure, as well as logistical issues which include licensure, credentialing and malpractice have been presented as barriers to the adoption of RPM interventions^53^. Another peripheral issue which may impede the adoption of RPM intervention is the challenge of data sharing. Effective implementation of RPM requires integration and consolidation of data from various devices and sources and making them easily available to the practitioners without infringing privacy laws or code of conduct^1,24^. Equity-related barriers that are associated with the affordability of technology, poor internet connectivity and poor health literacy among certain segments of the population should also be considered^54^. In addition, cultural considerations, which include a preference for personal touch and face-to-face contact among the population may also hinder the fast adoption of RPM intervention. A review of technology adoption for older people has demonstrated that technology adoption in the healthcare and long-term care settings needs to account for ethical issues such as autonomy/independence, social connectedness and human interactions, objectification, deception and social justice issues^55^. These are complex issues that require constant reflection on their use. Hence, healthcare practitioners will need to incorporate transparent audit mechanisms when deploying various RPM interventions to minimise their risks and unintended consequences. Policies and best practices around the deployment of these technologies should constantly be improved and amended from time to time to guide safe, responsible and ethical use of RPM interventions in the healthcare setting.

This review found that RPM interventions have increasingly been adopted in various clinical contexts in the past few years. This phenomenon is partially fuelled by the COVID-19 pandemic as well as the lack of healthcare workers during this period. However, we found that the current evidence base is dominated by developed countries in the West. Many developing countries are still grappling with health access issues and would benefit from the incorporation of RPM interventions in the health systems. In addition, there is a dominance of cardiovascular-related conditions in RPM intervention use, while other disease groups are less explored.

As more countries embrace remote monitoring technologies amid the pandemic, further studies are essential to assess their safety, clinical impact, quality of life, and cost-effectiveness, particularly in ensuring patient safety and service quality across various diseases at minimal costs. Additionally, more research is required to identify which devices and specifications offer the greatest clinical value to specific patient groups^50^. Economically, further evaluations should explore RPM interventions’ cost-effectiveness and benefits, considering both direct savings like reduced hospital stays and indirect costs like lost income or caregiver burdens. Furthermore, the extent to which RPM interventions can be leveraged to bridge health service delivery gaps in developing countries is an important area that needs more exploration.

The review suggests that RPM interventions combining patient education with active monitoring, whether alert-driven or routine, may enhance patient outcomes, meriting deeper empirical investigation.

Lastly, studies on RPM interventions’ implementation facilitators and barriers across care settings are vital for effective knowledge transfer. Such insights that encompass both best practices and suboptimal lessons are especially useful for health systems that are contemplating to scale-up technologies to accelerate the deployment of technology as a tool to meet rising healthcare needs and health demands.

## Methods

We conducted a systematic review in accordance with the Preferred Reporting Items for Systematic Reviews and Meta-Analyses (PRISMA). The review was registered on the PROSPERO database (ID: 412195). The PRISMA checklist can be found in Supplementary Table 1.

### Search strategy

Searches were conducted between February to March 2023 and finalised by 9 March 2023 (Supplementary Table 2). We developed a comprehensive search string that aimed to identify randomised controlled trials (RCTs) examining the impacts of remote patient monitoring (RPM) technologies in the inpatient setting and during the immediate care transition period. Searches were limited to January 2000 to May 2023 to reflect modern advancements in the field of RPM interventions. Ongoing discussion among the authors resulted in the development of two search themes (see Table 5): RPM and related concepts as well as RCTs. A subject librarian was also consulted to refine the search strings. After the finalisation of the search strings, a systematic database search was conducted on PubMed, CINAHL, PsycINFO, Embase and SCOPUS.

**Table 5:** Search strings developed for the systematic review

Concepts	Keywords and MeSH terms
#1 RPM and related concepts	(“biosensing techniques”[MeSH Terms] OR “Remote sensing technology”[MeSH] OR “remote sensing”[text word] OR “On body sensor”[text word] OR Biosensor*[text word] OR “Wearable device”[text word] OR “Constant health monitoring”[text word] OR “Wireless technology”[text word] OR “wearable sensor”[text word] OR “wearable”[text word] OR “medical sensor”[text word] OR “Body Sensor”[text word] OR “Passive monitor”[text word] OR “wireless monitor”[text word] OR “monitoring device”[text word] OR “wireless sensor”[text word]) (“Remote monitoring”[text word] OR “Remote patient monitoring”[text word] OR “self-monitoring”[text word] OR “self tracking”[text word] OR “remote tracking”[text word] OR “home monitoring”[text word] OR “wireless monitoring”[text word] OR “online monitoring”[text word] OR “online tracking”[text word] OR “telemonitoring”[text word] OR “ambulatory monitoring”[text word]) AND (“e-health”[text word] OR “m-health”[text word] OR “mobile”[text word] OR “mobile health”[text word] OR “telehealth”[text word] OR “telemedicine”[text word] OR “teleICU”[text word] OR “tele-ICU” [text word] OR “hospital at home”[text word] OR “digital health”[text word] OR “digital medicine”[text word] OR ((“smartphone”[MeSH Terms] OR “smartphone”[All Fields]) AND text[All Fields] AND word[All Fields]) OR “social network”[text word] OR “Web based”[text word] OR “online portal”[text word] OR “internet based”[text word] OR “cell phone”[text word] OR “mobile phone”[text word]) NOT (“self-monitoring”[text word] OR “self-management”[text word])
#2 RCT	(“Clinical Trial “[Publication Type] OR “Randomised Controlled Trial “[Publication Type] OR “randomised”[tiab] OR “placebo”[tiab] OR “therapy”[sh] OR randomly[tiab] OR trial[tiab] OR groups[tiab]) NOT (“animals”[MeSH] NOT “humans”[MeSH]).

### Inclusion and exclusion criteria

We also developed a set of inclusion and exclusion criteria to facilitate the screening of the abstracts and full texts (see Table 6).

**Table 6:** Inclusion and Exclusion Criteria

	Inclusion criteria	Exclusion criteria
Study design	• Studies employing an RCT design. • Studies assessing outcomes at the individual level (patient level or health workers level).	• Studies employing a non-RCT design. • Study protocols of RCTs. • Studies assessing impacts at the ecological level. • Studies without baseline measurement.
Data sources	• Studies published as full-text articles in English language peer-reviewed journal articles. • Conference proceedings.	• Non-English peer-reviewed journal articles • Book chapters, reports or other non-academic grey literature.
Population of interest	• Studies conducted on human populations admitted to the hospitals or during the immediate care transition period post-admission focusing on immediate transitory needs such as self-care and symptom management.	• Studies conducted on human populations in the outpatient or community setting and not immediately discharged from an inpatient setting and focus on long-term self-management.
Exposures/ Interventions	• Studies assessing the impacts of remote care monitoring in the inpatient or immediate care transition period looking at the following outcomes: (i) clinical outcomes, (ii) patient safety, (iii) quality of life, and (iv) cost outcomes.	• Studies assessing the impacts of tele-consultation or telemedicine on patients solely it the outpatient setting. • Studies assessing lifestyle modification interventions or self-management interventions. • No element of RPM intervention.
Time	• Studies that include measurements for at least two-time points (baseline and post-intervention).	• Studies that do not include measurements for at least two time points (baseline and post-intervention).

### Data extraction and selection process

A data extraction framework that includes information such as author/year, aim of the study, sample size, participation demographics, intervention components, duration of the intervention, RPM technology deployed and the outcomes examined (clinical outcomes, patient safety, quality of life and cost outcomes) was constructed. This framework was co-developed among all the authors through ongoing discussion until a unanimous agreement was reached. Three authors (S.Y.T., J.S., Y.W.) were involved in the data extraction process, and information was charted and documented in a spreadsheet. The authors subsequently cross-checked a random selection of papers to ensure consistency and relevance of the data extracted based on the framework.

### Critical appraisal

We employ the ‘revised Cochrane risk-of-bias tool for randomised trials (RoB 2) to assess the risk of bias of all the RCTs included in the synthesis. RoB 2 is structured to accommodate five domains of bias (risk of bias arising from the randomisation process, risk of bias due to deviations from the intended interventions, risk of bias due to missing outcome data, risk of bias in measurement of the outcome, risk of bias in selection of the reported result). In each domain, signalling questions were posed to guide the appraisals of different features of the trials. The responses to each signalling question ranged from yes (Y), partially yes (PY), partially no (PN), no (N), to no information (NI). Based on the responses to the signalling questions, bias is determined for each domain. Each domain can be judged as having ‘low’ risk of bias, ‘high’ risk of bias or ‘having some concerns’^56^.

### Data synthesis

Due to substantial heterogeneity in the populations of interest and primary outcomes reported, we were unable to perform a meta-analysis. Instead, we opted for a narrative synthesis approach by anchoring to the idea of the thematic synthesis approach proposed by Thomas and Harden^57^. A thematic synthesis approach entailed, firstly, conducting line-by-line coding of the relevant findings section extracted from the included studies by paying specific attention to the RPM interventions, intervention components and primary outcomes reported in these studies. This is followed by the development of the descriptive sub-themes from these narratives by identifying similarities and differences in the data patterns. These sub-themes then led to the construction of four key analytical themes that are centred on the RPM intervention features and the various impacts resulting from the deployment of these technologies. The first author led the analysis assisted by the third author. The second author audited and cross-checked the analysis, to ensure consistency, coherence and logic were adhered to. The entire data synthesis process underwent several rounds of discussion and iterations until all the authors were satisfied with the analytical themes that captured the functions and applications of all the RPM interventions documented as well as the impacts.

### Supplementary information

Supplementary Information
