# Supplementary material for: A systematic review of the impacts of remote patient monitoring (RPM) interventions on safety, adherence, quality-of-life and cost-related outcomes
Source: NPJ Digit Med. 2024 Jul 18;7:192. doi: 10.1038/s41746-024-01182-w (PMC11258279; doi:10.1038/s41746-024-01182-w)
Supplement: Supplementary file 1 — Supplementary Information [file 41746_2024_1182_MOESM1_ESM.pdf]

Supplementary Table 1: Data searches

**Database searches**
**(i) CINAHL** Searched on 9 March 2023)<br><br>("biosensing techniques" OR "Remote sensing technology" OR "Cell Phone" OR “remote sensing” OR “body sensor*” OR “Biosensor*” OR “Wearable device” OR “Wireless technology” OR “wearable” OR “medical sensor” OR “Body Sensor” OR “Passive monitor*” OR “wireless monitor*” OR “monitoring device*” OR “wireless sensor*” OR “smartphone” OR “mobile phone” OR “social network” OR “Web based” OR “online portal” OR “internet based”) AND ("telemedicine" OR “e-health” OR “m-health” OR “mobile health” OR “telehealth” OR “digital health” OR “digital medicine”) AND (“Monitor*” OR “telemonitoring” OR “online tracking” OR “remote tracking” OR “self tracking” OR “self- tracking”)<br><br>Limiters:<br><br>1. 1) Randomized Controlled Trial<br>2. 2) January 2000 till March 2023<br>3. 3) English language<br><br>4) All-text<br><br>**Total Hits: 346**<br><br>**(ii) EMBASE (**Searched on 9 March 2023)<br><br>('remote patient monitoring'/exp OR 'remote patient monitoring' OR (remote AND ('patient'/exp OR patient) AND ('monitoring'/exp OR monitoring))) AND ('biosensing techniques':ti,ab,kw OR 'remote sensing technology':ti,ab,kw OR 'cell phone':ti,ab,kw OR 'remote sensing':ti,ab,kw OR 'body sensor*':ti,ab,kw OR 'biosensor*':ti,ab,kw OR 'wearable device':ti,ab,kw OR 'wireless technology':ti,ab,kw OR 'wearable':ti,ab,kw OR 'medical sensor':ti,ab,kw OR 'body sensor':ti,ab,kw OR 'passive monitor*':ti,ab,kw OR 'wireless monitor*':ti,ab,kw OR 'monitoring device*':ti,ab,kw OR 'wireless sensor*':ti,ab,kw OR 'smartphone':ti,ab,kw OR 'mobile phone':ti,ab,kw OR 'social network':ti,ab,kw OR 'web based':ti,ab,kw OR 'online portal':ti,ab,kw OR 'internet based':ti,ab,kw) AND ('telemedicine':ti,ab,kw OR 'e-health':ti,ab,kw OR 'm-health':ti,ab,kw OR 'mobile health':ti,ab,kw OR 'telehealth':ti,ab,kw OR 'digital health':ti,ab,kw OR 'digital medicine':ti,ab,kw) AND ('monitor*':ti,ab,kw OR 'telemonitoring':ti,ab,kw OR 'online tracking':ti,ab,kw OR 'remote tracking':ti,ab,kw OR 'self tracking':ti,ab,kw OR 'self-tracking':ti,ab,kw) AND ([controlled clinical trial]/lim OR [randomized controlled trial]/lim) AND [2000-2023]/py<br><br>**Total Hits: 98**<br><br>**(iii) PsycInfo (Ovid) (**Revised search on 9th March 2023)<br><br>#1 ((biosensing techniques or remote sensing technology or cell phone or remote sensing or body sensor* or biosensor* or wearable device or wireless technology or wearable or

medical sensor or body Sensor or passive monitor* or wireless monitor* or monitoring device* or wireless sensor* or smartphone or mobile phone or social network or web based or online portal or internet based) and (telemedicine or e-health or m-health or mobile health or telehealth or digital health or digital medicine) and (monitor* or telemonitoring or online tracking or remote tracking or self tracking or self-tracking)).mp. [mp=title, abstract, heading word, table of contents, key concepts, original title, tests & measures, mesh word]

#2 limit 1 to ("0300 clinical trial" and yr="2000 -Current")

Total Hits: 85

(iv) PubMed (Searched on 9 March 2023)

((("biosensing techniques"[MeSH Terms] OR "Remote sensing technology"[MeSH] OR "Cell Phone"[Mesh]) OR (remote sensing[Title/Abstract] OR body sensor*[Title/Abstract] OR Biosensor*[Title/Abstract] OR Wearable device[Title/Abstract] OR health monitoring[Title/Abstract] OR Wireless technology[Title/Abstract] OR wearable[Title/Abstract] OR medical sensor[Title/Abstract] OR Body Sensor[Title/Abstract] OR Passive monitor*[Title/Abstract] OR wireless monitor*[Title/Abstract] OR monitoring device*[Title/Abstract] OR wireless sensor*[Title/Abstract] OR smartphone[Title/Abstract] OR mobile phone[Title/Abstract] OR cell phone[Title/Abstract] OR social network[Title/Abstract] OR Web based[Title/Abstract] OR online portal[Title/Abstract] OR internet based[Title/Abstract])) AND (("Telemedicine"[Mesh]) OR (e-health[Title/Abstract] OR m-health[Title/Abstract] OR mobile health[Title/Abstract] OR telehealth[Title/Abstract] OR telemedicine[Title/Abstract] OR digital health[Title/Abstract] OR digital medicine[Title/Abstract]))) AND (("Monitoring, Physiologic"[Mesh]) OR (Monitor*[Title/Abstract] OR telemonitoring[Title/Abstract] OR online tracking[Title/Abstract] OR remote tracking[Title/Abstract] OR self tracking[Title/Abstract]))

Filters: Clinical trial, randomized controlled trial

Total hits: 509

(v) Scopus (Searched on 9 March 2023)

(TITLE-ABS-KEY ("remote monitoring" OR "remote patient monitoring" OR "remote tracking" OR "home monitoring" OR "wireless monitoring" OR "online monitoring" OR "online tracking" OR "telemonitoring" OR "ambulatory monitoring") AND TITLE-ABS-KEY ("e-health" OR "m-health" OR "mobile" OR "mobile health" OR "telehealth" OR "telemedicine" OR "teleicu" OR "tele-icu" OR "hospital at home" OR "digital health" OR "digital medicine") OR TITLE-ABS-KEY ("biosensing techniques" OR "remote sensing technology" OR "remote sensing" OR "on body

sensor" OR "biosensor*" OR "wearable device" OR "constant health monitoring" OR "wireless technology" OR "wearable sensor" OR "wearable" OR "medical

sensor" OR "body sensor" OR "passive monitor" OR "wireless monitor" OR "monitoring device" OR "wireless sensor") OR TITLE-ABS-KEY ("social network" OR "web based" OR "online portal" OR "internet based" OR "cell phone" OR "mobile phone") AND

NOT TITLE-ABS-KEY ("self-monitoring" OR "self monitoring" OR "self-management" OR "self management") AND TITLE-ABS-KEY (("clinical trial" OR "randomized controlled trial" OR "randomized" OR "placebo" OR "randomly" OR "trial" OR "groups"))) AND PU BYEAR > 1999 AND PUBYEAR < 2024 AND (LIMIT-

TO (DOCTYPE , "ar") OR LIMIT-TO (DOCTYPE , "cp")) AND (LIMIT-TO (LANGUAGE , "english")) AND (LIMIT-TO (SUBJAREA , "comp") OR LIMIT-TO (SUBJAREA , "engi") OR LIMIT-TO (SUBJAREA , "medi") OR LIMIT-TO (SUBJAREA , "heal") OR LIMIT-TO (SUBJAREA , "deci") OR LIMIT-TO (SUBJAREA , "soci") OR LIMIT-TO (SUBJAREA , "psyc") OR LIMIT-TO (SUBJAREA , "arts") OR LIMIT-TO (SUBJAREA , "econ") OR LIMIT-TO (SUBJAREA , "nurs") OR LIMIT-TO (SUBJAREA , "mult")) AND (LIMIT-TO (SRCTYPE , "j") OR LIMIT-TO (SRCTYPE , "p"))

Total hits: 1,568

Supplementary Table 2: PRISMA checklist

Section and Topic		Item #	Checklist item	Location where item is reported
	**TITLE**			
Title		1	Identify the report as a literature review.	Title
	**ABSTRACT**			
Abstract		2	Provide a structured summary including, as applicable: background; objectives; data sources; study eligibility criteria, participants, and interventions; study appraisal and synthesis methods; results; limitations; conclusions and implications of key findings. See the PRISMA 2020 for Abstracts checklist for the complete list.	Abstract
	**INTRODUCTION**			
Rationale		3	Describe the rationale for the review in the context of existing knowledge, i.e., what is already known about your topic.	Section 1.0 Introduction
Objectives		4	Provide an explicit statement of the objective(s) or question(s) the review addresses with reference to participants, interventions, comparisons, outcomes, and study design (PICOS).	Section 1.0 Introduction
	**METHODS**			
Eligibility criteria		5	Specify the inclusion and exclusion criteria for the review and how studies were grouped for the syntheses with study characteristics (e.g., PICOS, length of follow-up) and report characteristics (e.g., years considered, language, publication status) used as criteria for eligibility, giving rationale.	Section 4.2 Inclusion and Exclusion Criteria
Information sources		6	Specify all databases, registers, websites, organisations, reference lists and other sources searched or consulted to identify studies. Specify the date when each source was last searched or consulted.	Section 4.1 Search Strategy
Search strategy		7	Present the full search strategies for all databases, registers and websites, including any filters and limits used.	Section 4.1 Search Strategy
Selection process		8	State the process for selecting studies (i.e., screening, eligibility). Specify the methods used to decide whether a study met the inclusion criteria of the review, including how many reviewers screened each record and each report retrieved, whether they worked independently, and if applicable, details of automation tools used in the process.	Section 4.3 Data Extraction and Selection Process
Study risk of bias assessment		11	Specify the methods used to assess risk of bias in the included studies, including details of the tool(s) used, how many reviewers assessed each study and whether they worked independently, and if applicable, details of automation tools used in the process.	Section 4.4 Critical Appraisal
	**RESULTS**			
Study selection		16a	Describe the results of the search and selection process, from the number of records identified in the search to the number of studies included in the review, ideally using a flow diagram.	Figure 1 PRISMA Flow Diagram of the Literature Search and Selection Process
		16b	Cite studies that might appear to meet the inclusion criteria, but which were excluded, and explain why they were excluded.	Not Applicable

Section and Topic		Item #	Checklist item	Location where item is reported
Study characteristics		17	Cite each included study and present its characteristics (e.g., study size, PICOS, follow-up period).	Section 2.1 Contexts and Characteristics of the Included Studies
Risk of bias in studies		18	Present assessments of risk of bias for each included study.	Section 2.2 Risk of Bias Assessment
Results of individual studies		19	For all outcomes, present, for each study: (a) summary statistics for each group (where appropriate) and (b) an effect estimate and its precision (e.g. confidence/credible interval), ideally using structured tables or plots.	Section 2.3 RPM Technologies Deployed<br>Section 2.4 Impacts of the RPM Interventions on Patient Safety<br>Section 2.5 Impacts of the RPM Interventions on Adherence<br>Section 2.6 Impacts of the RPM Interventions on Clinical Outcomes and Quality of Life<br>Section 2.7 Impacts of the RPM Interventions on Cost-Related Outcomes
	**DISCUSSION**			
Discussion		23a	Provide a general interpretation of the results in the context of other evidence.	Section 3.1 Summary of the Findings
		23b	Discuss any limitations of the evidence included in the review.	Section 3.2 Strength and Limitation of the Review
		23c	Discuss any limitations of the review processes used.	Section 3.2 Strength and Limitation of the Review
		23d	Discuss implications of the results for practice, policy, and future research.	Section 3.3 Implications for Policy and Practice
	**OTHER INFORMATION**			
Registration and protocol		24a	Provide registration information for the review, including register name and registration number, or state that the review was not registered.	Section 4.0 Methods
		24b	Indicate where the review protocol can be accessed, or state that a protocol was not prepared.	Section 4.0 Methods
		24c	Describe and explain any amendments to information provided at registration or in the protocol.	Not Applicable
Support		25	Describe sources of financial or non-financial support for the review, and the role of the funders or sponsors in the review.	Funding
Competing interests		26	Declare any competing interests of review authors.	Competing Interests

Section and Topic		Item #	Checklist item	Location where item is reported
Availability of data, code, and other materials		27	Report which of the following are publicly available and where they can be found: template data collection forms; data extracted from included studies; data used for all analyses; analytic code; any other materials used in the review.	Availability of Data, Code and Other Materials
